# Preparation of pH-Indicative and Flame-Retardant Nanocomposite Films for Smart Packaging Applications

**DOI:** 10.3390/s20195462

**Published:** 2020-09-23

**Authors:** Nedal Abu-Thabit, Abbas Saeed Hakeem, Khaled Mezghani, Elaref Ratemi, Mohamed Elzagheid, Yunusa Umar, Adhi Primartomo, Sirhan Al Batty, Abdul Kalam Azad, Sami Al Anazi, Ayman Ahmad

**Affiliations:** 1 Department of Chemical and Process Engineering Technology, Jubail Industrial College, Jubail Industrial City 31961, Saudi Arabia; Ratemi_e@jic.edu.sa (E.R.); elzagheid_m@jic.edu.sa (M.E.); Umar_y@jic.edu.sa (Y.U.); batty_sa@jic.edu.sa (S.A.B.); azad_a@jic.edu.sa (A.K.A.); anazi_sa@jic.edu.sa (S.A.A.); Mohammad_aa@jic.edu.sa (A.A.); 2 Center of Excellence in Nanotechnology (CENT), King Fahd University of Petroleum and Minerals (KFUPM), Dhahran 31261, Saudi Arabia; ashakeem@kfupm.edu.sa; 3 Mechanical Engineering Department, King Fahd University of Petroleum and Minerals, Dhahran 31261, Saudi Arabia; mezghani@kfupm.edu.sa; 4 Department of Mechanical and Manufacturing Engineering Technology, Jubail Industrial College, Jubail Industrial City 31961, Saudi Arabia; primartomo_a@jic.edu.sa

**Keywords:** sensor, pH, colorimetric, food, packaging, nanocomposite, flame-retardant, nanoclay, PVA

## Abstract

There is an increasing demand for sustainable and safe packaging technologies to improve consumer satisfaction, reduce food loss during storage and transportation, and track the quality status of food throughout its distribution. This study reports the fabrication of colorimetric pH-indicative and flame-retardant nanocomposite films (NCFs) based on polyvinyl alcohol (PVA) and nanoclays for smart and safe food packaging applications. Tough, flexible, and transparent NCFs were obtained using 15% nanoclay loading (PVA-15) with superior properties, including low solubility/swelling in water and high thermal stability with flame-retardant behavior. The NCFs showed average mechanical properties that are comparable to commercial films for packaging applications. The color parameters were recorded at different pH values and the prepared NCFs showed distinctive colorimetric pH-responsive behavior during the transition from acidic to alkaline medium with high values for the calculated color difference (∆E ≈ 50). The prepared NCFs provided an effective way to detect the spoilage of the shrimp samples via monitoring the color change of the NCFs during the storage period. The current study proposes the prepared NCFs as renewable candidates for smart food packaging featuring colorimetric pH-sensing for monitoring food freshness as well as a safer alternative choice for applications that demand films with fire-retardant properties.

## 1. Introduction

Proper packaging of processed foods, fresh vegetables, and fruits save them from spoilage during transportation and storage. Examples of potential spoilage include: microbial spoilage, oxidation, and moisture changes due to improper handling, which leads to waste [1,2]. Health issues can also arise from decayed foods, fruits, and vegetables and sometimes lead to illnesses and/or toxicity [3,4]. One of the advantages of packaging processed foods is to enable the safe transportation of the foods from the point of origin to the point and time of consumption [5,6,7,8]. To attract industry requirements and satisfy consumer desire, food packaging should be convenient and cost-effective, while maintaining food quality, safety, and environmental sustainability [9]. The development of new materials, particularly innovative biopolymer formulations, must fulfill the aforementioned requirements. The availability, biodegradability, and unique properties of biopolymers make their use in multiple food-packaging applications superior [10]. One of the most challenging trends in the development of innovative biopolymers is to obtain them from agricultural commodities, natural additives, and/or agricultural wastes [11,12,13]. From the food industry standpoint, concerns such as the safety and risks associated with these new additives, migration properties, and possible human ingestion and regulations need to be considered [14,15]. The introduction of natural active additives to packaging materials not only resolves the safety concern but also provides significant advantages compared to the direct addition to food, such as the lower amount of active substances required, controlled release to food, and elimination of additional steps during processing [16].

Color, as an indicator, has an important role in the acceptability of foods. Colorants are being in use to ensure uniformity in food and also as an indicator of food quality. Synthetic colorants have always been under question regarding their safety. Therefore, consumers prefer natural colorants to synthetic ones [17]. As a result, interest in natural colorants has increased because of the apparent lack of toxicity [18]. Among other promising natural colorants are anthocyanins [19]. Due to their non-toxicity, water-solubility, visibility to the human eye, and colorimetric pH sensitivity, anthocyanin-rich extracts has increasingly attracted the food industry as a replacement for the synthetic pH indicators, such as methyl red, cresol red, bromocresol green, bromocresol purple, chlorophenol, bromothymol blue, and xylenol [19,20,21]. Their red-orange to blue-violet pigments are present in many fruits and vegetables such as red cabbage, red grapes, purple corn, strawberry, blackberry, eggplant, and black olives [19]. Anthocyanins extracted from red cabbage (*Brassica* sp.) is used widely around the world ranging from drink dye to food due to its fascinating deep blue and red color with broad pH sensitivity [22,23,24,25,26,27]. On the other hand, anthocyanins from other sources such as grape skin, and elderberry show only a reasonable degree of color at pH < 4 [28]. Anthocyanins’ red cabbage colors vary from red at low pH to blue and green at high pH and their use is therefore not limited to acidic products but can be extended to neutral products as well. A major concern lies in their instability during processing and storage [29]. To overcome this, solid supporters have been incorporated with anthocyanins within their packaging films [30,31]. Bio-based materials derived from natural sources are considered potential substitutes for conventional plastic materials because they are biodegradable, cost-effective, and widely available [13,26,27]. However, the properties of those films must be improved if they are intended to compete with petroleum-based products, especially mechanical properties and water affinity. Several studies researched the use of bio-based or biodegradable polymers and anthocyanins from red cabbage, grape and spinach extracts to produce biodegradable pH-indicative films for visual monitoring of food freshness [23,24,32,33,34].

Due to its non-toxicity, biodegradability, biocompatibility, high transparency, and mechanical properties, polyvinyl alcohol (PVA) is one of the most investigated polymers in different areas including packaging materials, adhesives, furnishings and textile industries [35]. The key limitations for using PVA films in food packaging applications are their water solubility, high water-swelling, and flammability. The high swelling and solubility of PVA films limit their mechanical integrity during their application period, while their flammability is considered as a potential hazardous during storage/use in the above-mentioned applications. Regardless, researchers have investigated different techniques to reduce the solubility/swelling of PVA films including blending with hydrophilic polymers, graft copolymerization and chemical modification [36,37,38,39,40]. One of the most interesting methods to prepare multifunctional PVA nanocomposites/bionanocomposites is by blending with different inorganic/organic nanomaterials such as nanoclay [41], silver nanoparticles [42,43,44], cellulose nanofibrils [45,46], chitosan nanoparticles [47], and lignin nanoparticles [48]. Due to their excellent barrier properties combined with good transparency, naturally occurring nanoclays are considered as ideal, renewable and green nanomaterials for packaging applications [49]. Nanoclays have been used as a reinforcing matrix to produce PVA-based biodegradable nanocomposites with superior thermal, mechanical, and barrier properties [50,51,52,53,54,55,56,57,58]. 

The flammability of the commercial thermoplastics has been eliminated/reduced by incorporating flame-retardant additives (e.g., halogenated, phosphorus or inorganic compounds) and nanomaterials (e.g., nanoclays, carbon nanotubes, silica nanoparticles (NPs), metal oxides, NPs and Polyhedral Oligomeric SilSesquioxane NPs) [59]. Halogen flame-retardants have been phased out due to their toxicity arising from the formation of toxic compounds/gases during burning/degradation, corrosion as well as their bioaccumulation in animals and humans, which is associated with numerous health effects such as endocrine disruption, reproductive toxicity, and cancer [60,61]. The effective use of phosphorous or inorganic compounds requires high percentage loadings into the polymer matrix in the range of 30–60% [62,63,64,65,66], which can lead to loss of mechanical properties [67] as well as product discoloration during their application period [68]. In comparison, nanomaterials-based intumescent nanocomposites require much lower loadings in the range of 0.5–25% [69] without significant deterioration of the mechanical properties for the resulted nanocomposite systems. This could be attributed to their high surface area and the difference in flame-retardant action mechanism [61,70]. Nanoclays are effective for fabricating flame-retardant nanostructured platforms and coatings [71,72,73,74] and flame-retardant nanocomposites [75,76]. This can be recognized from the commercially available two products based on synergistic enhancements of clay nanocomposites for fire safety applications, which are: (1) a Wire & Cable jacket material (organoclay + aluminum hydroxide) produced by Kabelwerk Eupen AG; and (2) a series of polypropylene + organoclay + flame-retardant systems (Maxxam™ FR) produced by PolyOne^®^ [77].

**Figure 1:**
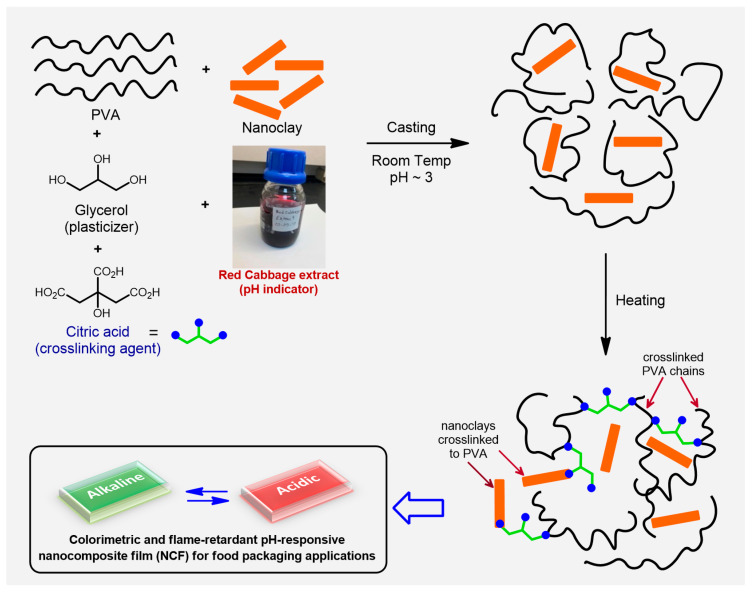
Schematic illustration for the preparation process of the pH-responsive nanocomposite films (NCFs).

As illustrated in (Figure 1), the current study reports the preparation of pH-indicative and flame-retardant nanocomposite films (NCFs) using PVA as the host polymer, nanoclays as the reinforcing materials, citric acid as the crosslinking agent, glycerol as a plasticizer, and anthocyanin extracted from red cabbage as a natural pH-indicator. To the best of our knowledge, this is the first study that reports the preparation of PVA/nanoclays nanocomposites targeting their use as pH-indicative/flame-retardant NCFs for food packaging applications. 

## 2. Experimental

### 2.1. Materials and Methods

Polyvinyl alcohol (PVA) (98–99% hydrolyzed, low molecular weight) was acquired from Alfa Aesar (Kandel, Germany); Nanoclay–hydrophilic bentonite, particle size ≈ 25 μm was acquired from Aldrich (Milwaukee, WI, USA). All other chemicals and reagents were used as received. Red Cabbage was purchased from local stores. The pH buffers were prepared based on a PBS buffer system as described in our previous work [78]. 

### 2.2. Preparation of the Nanocomposite Films (NCFs)

The pH-responsive nanocomposite films (NCFs) were fabricated with four different recipes containing 0, 5, 15 and 25% nanoclays denoted as nanocomposite PVA-0, PVA-5, PVA-15 and PVA-25, respectively. The film PVA-0 represents the neat PVA film without added nanoclay or other reagents. The fabrication steps were carried out as follows: Ten grams of PVA were dissolved in 90 g distilled water by heating at 60 °C with continuous stirring until dissolved completely. Red cabbage extract was prepared soaking the chopped red cabbage (≈130 g) in a solution made from 150 mL ethanol, 150 mL water and 4 mL concentrated HCl. The mixture was sonicated for 20 min, and the extracted solution was filtered off and stored in a glass bottle for subsequent use. The preparation of the NCFs was carried out as illustrated in (Figure 1). In brief, 10 mL of 10% PVA solution was mixed with red cabbage extract (5 mL), nanoclay (5, 10 or 15% wt./wt.), glycerol (0.10 g) and citric acid (5, 10, or 20% wt./wt.). The solution was stirred for 2 h and the pH was adjusted to ≈3, with the help of NaOH and HCl solutions. After that, the solution was sonicated for 15–20 min, and then the solution was cast on glass Petri dishes at room temperature for 48 h. After that, the dried films were peeled off and crosslinked immediately at a predetermined temperature (135 °C or 150 °C) for a specified time in the range of 5–45 min. The crosslinked NCFs were immersed into deionized water at room temperature for 12 h to stabilize the NCFs and to remove the excess unreacted citric acid and the non-crosslinked PVA. Finally, the NCFs were dried at 50 °C with an average thickness of 100 μm. The NCFs prepared for mechanical analysis were cast with dimensions of 14 cm × 14 cm and thickness of ≈0.5 mm. 

### 2.3. Characterization of the NCFs

#### 2.3.1. Water Swelling

Water swelling was measured according to Abdullah and DONG [58]. Square samples of all NCFs in a size of 2 × 2 cm^2^ were pre-dried in a vacuum oven at 50 °C for 24 h and then cooled to room temperature in a desiccator prior to weighing them as initial dry weight (*W_i_*). After that, the dried NCF samples were immersed in 100 mL distilled water at room temperature for 24 h to reach an equilibrium state. The NCFs were removed from water and their surfaces were gently wiped with tissue paper. The final weights of the swollen NCFs after immersion were measured and denoted as (*W_f_*). Three samples for each type of NCF have been tested along with reported average data and associated standard deviations shown as error bars in the respected graphs. The percentage water swelling (*SW*) was calculated according to Equation (1):

(1) $$SW\;(\%)\;=\;\frac{W_{f}\;-\;W_{i}}{W_{i}}\;\times 100\%$$

#### 2.3.2. Water Solubility 

Water solubility was measured according to Abdullah and Dong [58]. All swollen samples from the above water swelling tests were used to calculate the water solubility of the NCFs. The swollen samples were dried again in a vacuum oven at 50 °C for 24 h, followed by cooling to room temperature in a desiccator for 30 min. Finally, the NCF samples were weighed to acquire the dry weight after immersion (*W_d_*). Equation (2) was used to calculate the percentage of water solubility (S):

(2) $$S\;(\%)\;=\;\frac{W_{i}\;-\;W_{d}}{W_{i}}\;\times \;100\%$$

#### 2.3.3. FTIR Spectroscopy

Fourier transform infrared spectra of the NCFs were recorded using Thermo Scientific spectrometer (Madison, WI, USA), model (Nicolet 6700), operating with smart iTR^TM^ Attenuated Total Reflectance (ATR) accessory as sampling mode. Measurements were performed in a medium infrared range (4000–500 cm^−1^) with a spectral resolution of 4 cm^−1^ and 32 scans per spectrum.

#### 2.3.4. Thermal Analysis and Flame-Retardant Property of the NCFs

Thermogravimetric analysis (TGA) was carried out using Netzsch (Berlin, Germany), model (STA_449_F3). Around 5–8 mg of the film samples was heated in argon atmosphere from room temperature up to 800 °C with a heating rate of 10 °C/min. Differential scanning calorimetry (DSC) was carried out using Mettler Toledo (USA), model (DSC822e). Around 3–5 mg of the film samples was heated in argon atmosphere from room temperature up to 500 °C with a heating rate of 10 °C/min. The percentage degree of crystallinity (*χ*_*c*_) was calculated using Equation (3):

*χ_c_*_(%)_ = (∆*H_m_*/∆*H_f_*) × 100 (3)

where ∆*H_m_* = enthalpy of melting for the unknown film sample, and ∆*H_f_* = enthalpy of fusion for 100% crystalline PVA, which was reported to be 138.6 J/g [79].

The flame-retardant property of the NCFs was tested by the burn test and differential scanning calorimetry [48].

#### 2.3.5. Mechanical Analysis

Tensile tests were conducted according to ASTM D882 using Lloyds tensile instrument (West Sussex, UK) with a load cell of 100 N and a controlled rate of 50 mm/min. The sample size and shape were according to ASTM D638 type V. For each type of material, 5 samples were tested and the average values of the properties were reported. The tensile properties, such as the modulus of elasticity, tensile strength, and percent of elongation, were determined from the tensile plots. The modulus was obtained from the slope of the initial curve of the stress–strain plot.

#### 2.3.6. XRD Analysis

XRD analysis for the NCF samples was carried out using a Rigaku MiniFlex X-ray diffractometer (Tokyo, Japan) with Cu Kα1 radiation (γ = 0.15416 nm), a tube current of 10 mA, and an accelerating voltage of 30 kV with angle 2θ from 5° to 60°. The scanning rate was 2°/min with a step size 0.02°.

#### 2.3.7. SEM Analysis

The microstructure of the prepared NCF samples was examined with a scanning electron microscope Coxem (Daejeon, Korea), model CX200 Plus. The specimens were sputter coated with gold and the cross-sectional fracture surfaces were mounted vertically on 90° pin stubs with carbon tape and imaged using an accelerating voltage of 10–15 kV.

#### 2.3.8. Optical Properties of the Film

Light transmittance of the prepared NCFs was plotted as a function of wavelength in the range of 400–800 nm, using a double beam spectrophotometer Cintra 2020 (GBC Scientific Equipment, Melbourne, Australia). The color parameters (a*, b* and L*) of the NCFs were measured using colorimeter device manufactured by Sheen Instruments (Metamora, MI, USA), model No. 281 SPECTRO–GUIDE 45/0 with white background. The values of the rectangular coordinates (L*, a*, b*), where (L*) is lightness (from L = 0 for black to L = 100 for white), a* is the degree of redness or greenness (a = −60 for green to a = 60 for red), and b* is the degree of yellowness or blueness (from b = −60 blue to b = 60 for yellow) were recorded at different pH levels, and the total color differences, ∆E* was calculated by using Equation (4):

(4) $$∆E={\left[{\left(∆L*\right)}^{2}+{\left(∆a*\right)}^{2}+{\left(∆b*\right)}^{2}\right]}^{\frac{1}{2}}$$

where: ∆L* = L* − L0*; ∆a* = a* − a0*; ∆b* = b* − b0* (L0*, a0* and b0* are the color parameters of the reference NCF at specific pH level.

#### 2.3.9. Sensing Shrimp Spoilage

NCFs were evaluated as intelligent films for use in the packaging industry; shrimps with an average weight of 5 g were purchased from the local supermarket. About 15 g of shrimp and the testing NCF (prepared at pH 3) were sealed in a glass Petri dish using plastic film and the color change of the film was recorded every 6 h for a total period of 24 h.

#### 2.3.10. Statistical Analysis

The properties of the NCFs were measured with individually prepared films in triplicate. The results are provided as the mean ± SD (standard deviation) values. One-way analysis of variance (ANOVA) was performed and the significance of each mean property value was determined (*p* < 0.05) with Tukey’s HSD (honestly significant difference) test for paired comparison, using IBM^®^ SPSS^®^ statistics 20 software (IBM Co., New York City, NY, USA).

## 3. Results and Discussion

### 3.1. Optimization of Film Fabricating Parameters

**Figure 2:**
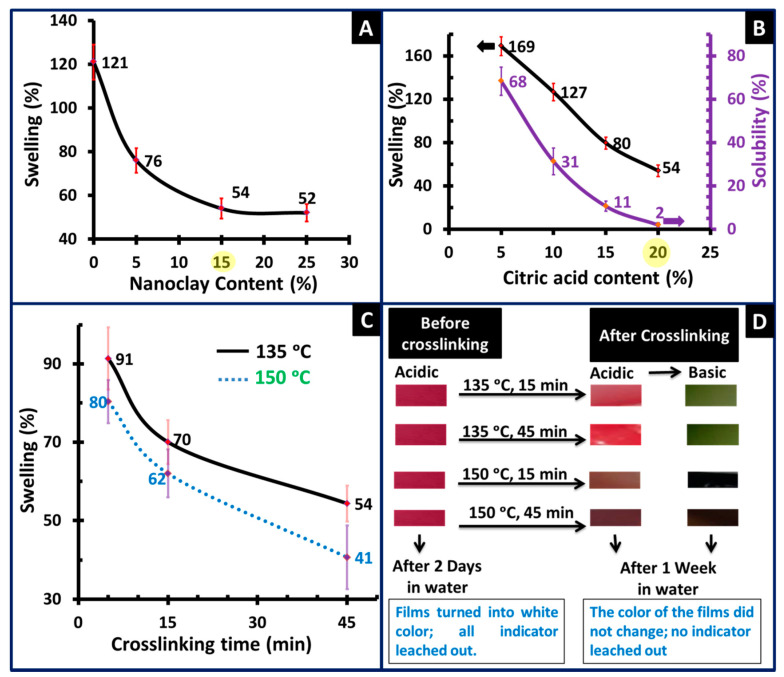
Optimization of parameters for fabricating the NCFs including: (**A**) the effect of nanoclay content on the swelling of the NCFs; (**B**) the effect of citric acid content on the swelling and solubility of the polyvinyl alcohol (PVA)-15 NCFs; (**C**) the effect of the crosslinking time and the crosslinking temperature on the swelling of the PVA-15 NCFs; (**D**) illustration of the color change and leaching out of the indicator from PVA-15 NCFs before and after various crosslinking treatments.

All NCFs were obtained without visual phase separation even at 25% loading of nanoclay (Figure 2A). It has been reported that bentonite nanoclays with 25% loading exhibited homogeneous dispersion in PVA polymer matrix [51]. The swelling of the NCFs decreased as the content of the nanoclay increased up to 15% and then the swelling leveled off to almost a constant value. The decrease in water swelling could be attributed to the non-swelling properties of the nanoclays as well as the increase in the hydrophobicity of the NCF (or reduced PVA hydrophilicity) from the added bentonaite nanoclays [52,80]. NCFs with 15% loading of nanoclay reached an acceptable swelling level of 52%, which is much lower than those reported for PVA/chitosan/HNTs NCFs crosslinked with gluteraldehyde which exhibited 137% swelling with 5% loading of nanoclay [81]. Different research groups have reported the preparation of crosslinked PVA films using citric acid with concentrations in the range of 10–50 wt.% [82,83,84,85]. However, Brick et al. reported that PVA films crosslinked with 40 wt.% citric acid, cured at 130 °C for 40 min, showed brittle behavior due to the excessive crosslinking density without any observed plastic deformation [85]. In the current study, the optimum concentration of the citric acid was investigated in terms of swelling and solubility of the NCFs in water (Figure 2B). It can be inferred that, as the concentration of citric acid increased, both swelling and solubility decreased due to the increased crosslinking density between different PVA chains. The highest dimensional stability of the NCFs was obtained at 20 wt.% citric acid content, which provided tough and flexible NCFs with 2% solubility and reasonable water swelling of 54% (Figure 2B). It is worth mentioning that the obtained NCFs were stable even in hot water up to 60–80 °C, which can be envisaged for other applications such as polyelectrolyte membranes for medium temperature alkaline fuel cells.

The NCFs were investigated in terms of (1) swelling of the NCFs; (2) color change of the crosslinked films; and (3) leaching out of the embedded indicator from the crosslinked NCFs. Truong et al. reported the optimum crosslinking parameters for PVA/citric acid with 20–30 wt.% citric acid content and crosslinking temperature of 130 °C for a total time of 45 min [82]. Similar optimum crosslinking parameters were reported by Brick et al. for crosslinking PVA/citric acid with 10 wt.% citric acid content at a temperature of 130 °C for a total time of 40 min [85]. In the current study, the cast NCFs were crosslinked at either 135 °C or 150 °C, for a total time of 5, 15, and 45 min (Figure 2C). The use of 150 °C was investigated to expedite the curing process of the NCFs. It should be noted that 150 °C is the highest crosslinking temperature that can be used as PVA matrix starts to degrade above this temperature [41]. As can be inferred from (Figure 2C), the swelling of the NCFs was reduced as the crosslinking time increased from 5 to 45 min at both temperatures. The optimum swelling was obtained using a crosslinking period of 45 min, with swelling values of 41% and 54% at 150 °C and 135 °C, respectively. However, it was observed that the NCFs crosslinked at 135 °C did not show significant color change after the crosslinking reaction, while the color of the NCFs crosslinked at 150 °C has changed from pink-reddish color to dark brown after the crosslinking reaction (Figure 2D). Hence, the optimum crosslinking temperature and time parameters were selected to be 135 °C for 45 min. Another observed advantage of the crosslinked films is the stability of the indicator inside the matrix of the NCFs. It was observed that the immobilized indicator did not leach out from the crosslinked NCFs, which is important for obtaining reusable films with a longer shelf life, especially in humid/wet environments (Figure 2D).

### 3.2. Characterization of the NCFs

#### 3.2.1. FTIR Analysis

**Figure 3:**
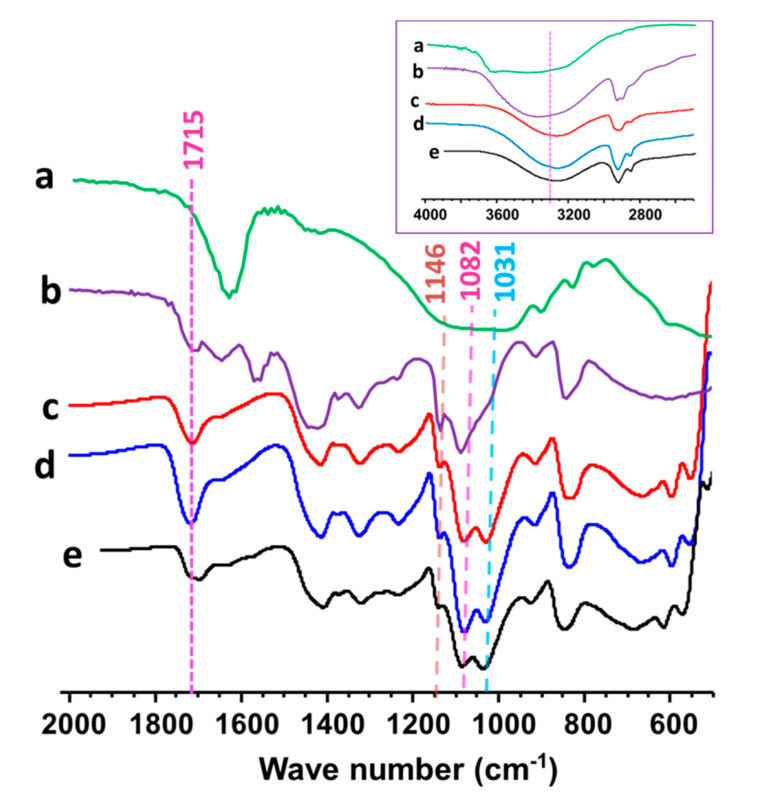
FTIR spectra in the range of 500–2000 cm^−1^ for (**a**) neat bentonite nanoclay, (**b**) neat PVA film, (**c**) PVA-15 crosslinked at 135 °C -15 min, (**d**) PVA-15 crosslinked at 135 °C -45 min, and (**e**) PVA-15 crosslinked at 150 °C -15 min. The inset image represents the FTIR spectral features in the range of 2600–4000 cm^−1^.

FTIR spectra for neat PVA film, neat bentonite nanoclay, and PVA-15 NCFs are displayed in Figure 3. The crosslinked films exhibit new peak at ≈1712–1717 cm^−1^ that is characteristic of carbonyl stretching of the ester functional groups [82] (Figure 3c–e). This demonstrates the successful esterification occurred between the carboxylic acid groups of citric acid and the hydroxyl moieties of PVA to form the crosslinked polymer films [82]. The effect of crosslinking temperature and time was investigated to evaluate the best conditions and to avoid possible degradation of the crosslinked films. The film crosslinked at 135 °C for 45 min exhibited the highest intensity at 1712–1717 cm^−1^ as compared to films crosslinked for 15 min at either 135 °C or 150 °C. These crosslinking parameters are similar to those reported for crosslinking of electrospun PVA membranes with different organic acids (130 °C and 30 min), which provided good film stability in water [82]. The crosslinked PVA-15 NCFs exhibit peaks at ≈1031 cm^−1^, which is attributed to the Si-O bonds of nanoclay [86]. The peak around 1146 cm^−1^ is assigned to the C-C bonds associated with the crystallization of PVA [86]. The interfacial crosslinking of PVA/bentonite nanoclays can be inferred from the present Si-O-C absorbance peak at 1082 cm^−1^ which verifies the covalent reaction between bentonite nanoclays and citric acid [86]. The intensity of the –OH band at ≈3200–3300 cm^−1^ was reduced after the crosslinking reaction due to the formation of the ester bonds, which decreases the number of available free hydroxyl groups tethered from PVA chains [83] (Figure 3). Hence, the FTIR analysis of the PVA-15 NCFs confirmed the successful crosslinking reaction at 135 °C for 45 min.

#### 3.2.2. Thermal Analysis

**Figure 4:**
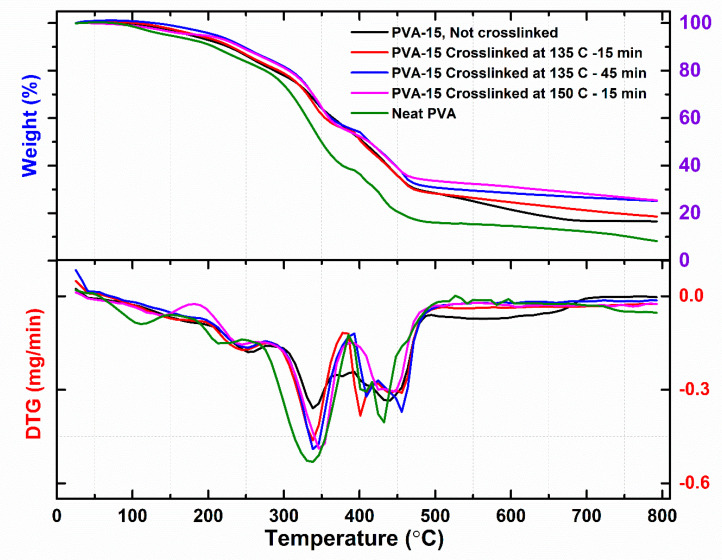
Thermogravimetric analysis (TGA) (**top**) and DTG (**bottom**) thermograms for various prepared NCFs.

**Figure 5:**
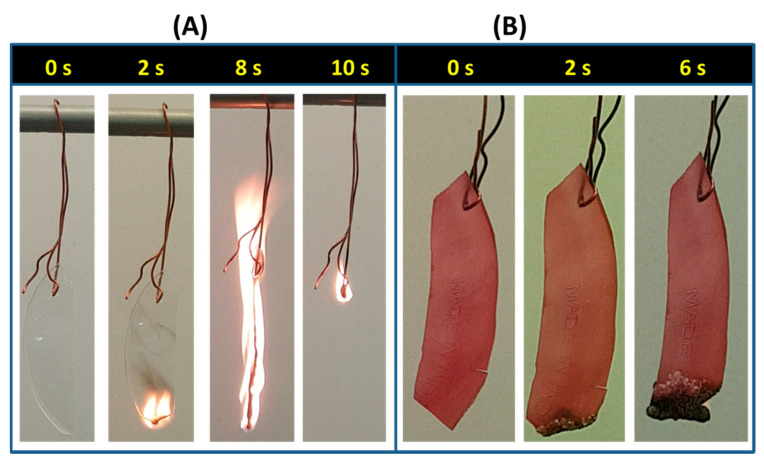
Burning test for the (**A**) neat PVA film, and (**B**) PVA-15 NCF crosslinked at 135 °C for 45 min.

Figure 4 shows the TGA and DTG curves for films of neat PVA, and PVA-15 with/without crosslinking treatments. It can be observed from TGA curves that all the crosslinked PVA-15 NCFs exhibit higher thermal stabilities than those of either neat PVA film or the non-crosslinked PVA-15 NCF. In the temperature range of 25–800 °C, all films exhibit four steps of weight loss. The first stage occurs in the temperature range of 40–150 °C, which is attributed to the loss of water molecule absorbed by the hydrophilic PVA matrix. It can be inferred that the crosslinked PVA-15 NCFs display lower absorbed water content compared to the neat PVA or non-crosslinked PVA-15 films, which could be attributed to (1) the increased hydrophobicity of the NCFs due to the presence of nanoclay; and (2) the crosslinking effect, which reduces the number of the available hydrophilic site (–OH groups) in the PVA polymer chains. The PVA-15 crosslinked at 135 °C for 45 min shows less than 2% weight loss up to 150 °C, which reflects the suitability of the used crosslinking parameters and the improved hydrophobicity of the NCF. The second stage falls in the temperature range of 150–380 °C, which is attributed to the loss of –OH groups and the deacetylation of PVA chains [87]. It is evident that the NCFs exhibit less degradation (38–45% weight loss) in this temperature range as compared to the neat PVA film (60% weight loss), which could be attributed to the incorporation of the nanoclays in the PVA matrix. PVA-15 crosslinked at 135 °C for 45 min showed the highest thermal stability with a weight loss of 38% in the second stage. The third stage occurs in the temperature range of 380–500 °C, which is attributed to the main chain degradation of the polymeric backbone [87]. The last degradation stage and breakdown of the charred residue occurs in the temperature range of 500–800 °C, which is attributed to the combustion of carbonaceous char residue. Neat PVA produced 8% charred residue which is comparable to the results obtained by Kaiyan and Anil for pristine PVA films (6% char residue) [53]. Indeed, both PVA-15 NCFs crosslinked either at 135 °C for 45 min, or at 150 °C for 15 min, produced the highest char residue of ≈25%. These results could be attributed to the flame-retardant effect of the nanoclays, which have been used for preparing fire retardant NCFs and intumescent coatings [69,88]. To demonstrate the flame-retardant behavior of the NCFs, burning tests were carried out for neat PVA film and PVA-15 NCF crosslinked at 145 °C for 45 min (Figure 5). It can be observed that neat PVA sustained a self-propagating flame after ignition, and no residue was left after the burning test (Figure 5A). In contrast, the flame of the PVA-15 NCF did not self-propagate even after 6 s. (Figure 5B). The fire-retardant property of the PVA-15 NCF could be attributed to the migration of the nanoclays to the surface of the polymer during the combustion process, leading to the formation of a physical barrier silicate layer, which slows mass and heat transfer, limits the oxygen flow and escape of volatiles [69].

**Figure 6:**
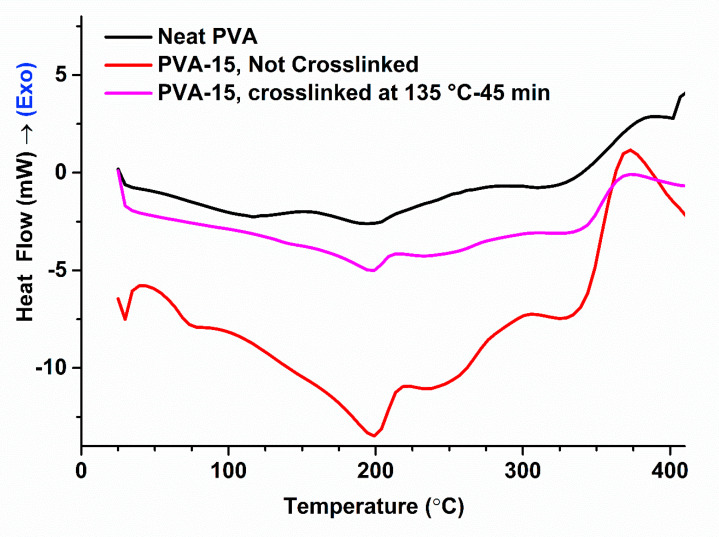
Differential scanning calorimetry (DSC) thermograms for the prepared NCFs.

DSC thermograms for the NCFs are displayed in Figure 6, and the analyzed results are summarized in Table 1. As can be seen, neat PVA film exhibits a glass transition temperature of T_g_ = 76 °C, an endothermic melting peak at T_m_ = 194 °C, and a corresponding crystallinity index of χ_c_ = 25%. It should be noted that the peak around 110 °C corresponds to the evaporation entropy for the physically bonded water molecules [89]. The former peak disappeared completely from the thermograms of the NCFs, which indicates their lower tendency to bind with water as well as their lower hydrophilicity compared to neat PVA film. PVA-15 NCFs showed two distinct and overlapping melting peaks one around the bulk (T_m1_ ≈ 199 °C) and another one at higher melting temperature (T_m2_ ≈ 233 °C), with similar behavior for the reported PVA/Na^+^ montmorillonite nanocomposites, which showed MMT-induced crystal phase at 235 °C [56]. The new T_m2_ endothermic peak is an indication for the presence of a new higher-T_m_ crystal phase, rather than a higher T_m_ morphology with bulk PVA structure [56]. For the non-crosslinked PVA-15 NCF, the slight increase in T_g_ and T_m_ values (T_g_ = 81 °C, T_m_ = 199 °C) and decreased crystallinity (χ_c_ = 18%) could be ascribed to the incorporation of nanoclays [54]. After the crosslinking reaction, PVA-15 NCF showed further reduction in the crystallinity index (χ_c_ = 10%) with no change in the T_m_ value. The lower crystallinity after the crosslinking reaction could be attributed to the restricted segmental motion of the molecules, which are commonly observed phenomena in most polymers [54]. The T_g_ value for the crosslinked PVA-15 NCF was too weak to be detected which could be attributed to the enhanced dispersion of nanoclay after crosslinking reaction, i.e., “neatly intercalated” nanocomposites, or it is suppressed due to the polymer confinement [56]. Similarly, by comparing the enthalpy of melting peak (ΔH_m1_), the crosslinked PVA-15 film showed the lowest ΔH_m1_ value among the NCFs, which provides evidence for the suppression of the melting in the crosslinked PVA-15 NCF due to the confinement of polymer chains in between clay platelets as well as to the layers structure, which provides an indication of the strong interaction between the polymer and clay [79]. Finally, the suppression of the melting enthalpy of the crosslinked PVA-15 film, the higher degradation temperature (T_d_ = 326 °C) as compared to the neat PVA film (T_d_ = 310 °C), and the lowest enthalpy of degradation (ΔH_d_) provide strong evidence for the flame-retardant behavior of the prepared NCFs [48] (Table 1).

### 3.3. XRD Analysis

**Figure 7:**
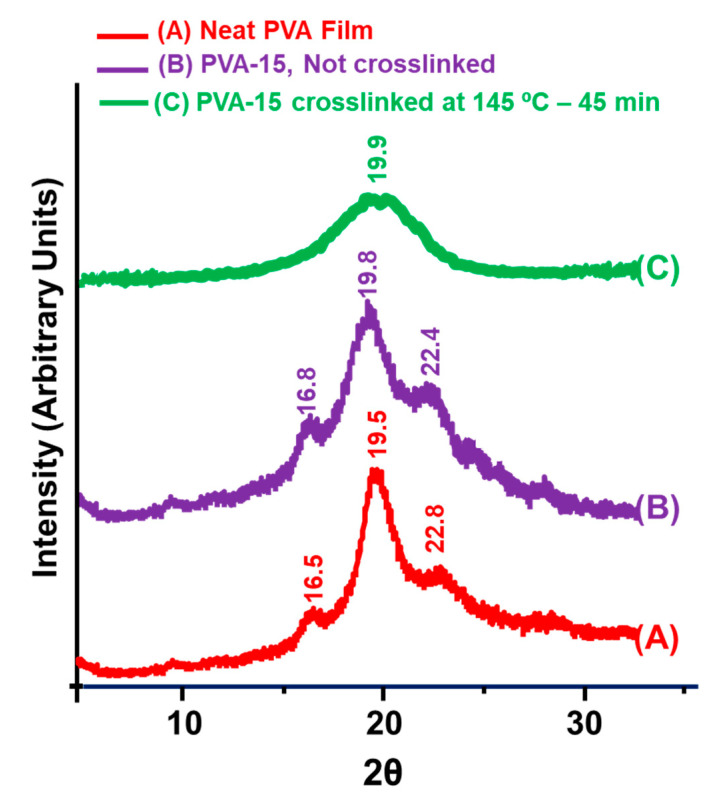
XRD analysis of the prepared NCFs.

The XRD spectrograms of the prepared NCFs are displayed in Figure 7. Neat PVA film exhibits main characteristic peaks at 2θ = 16.5°, 19.5° and 22.8°, which corresponds to 100, 10ī, and 200 crystalline reflection of monoclinic PVA crystal [56]. The non-crosslinked PVA-15 NCF showed similar peaks with less intensity and a slight shift of the main peak at 2θ = 19.8°, which could be attributed to the decreased crystallinity after incorporating the nanoclays. The shift in the main peak from 19.5° to 19.8° could be attributed to the presence of nanoclay, which is characterized by a reflection peak at 2θ = 19.8° [56]. After the crosslinking reaction with citric acid, the main peak at 2θ = 19.9° became broader with a further decrease in the intensity, while other peaks disappeared. This could be attributed to the reduced numbers of intramolecular hydrogen bonding after the crosslinking reaction, which converts the semicrystalline thermoplastic PVA into a more amorphous state with lower crystallinity [90]. Similar findings were reported for the PVA/20 wt.% MMT/10 wt.% polystyrenesulfonate NCFs crosslinked with 5 wt.% glutaraldehyde solution [55].

### 3.4. SEM Analysis

**Figure 8:**
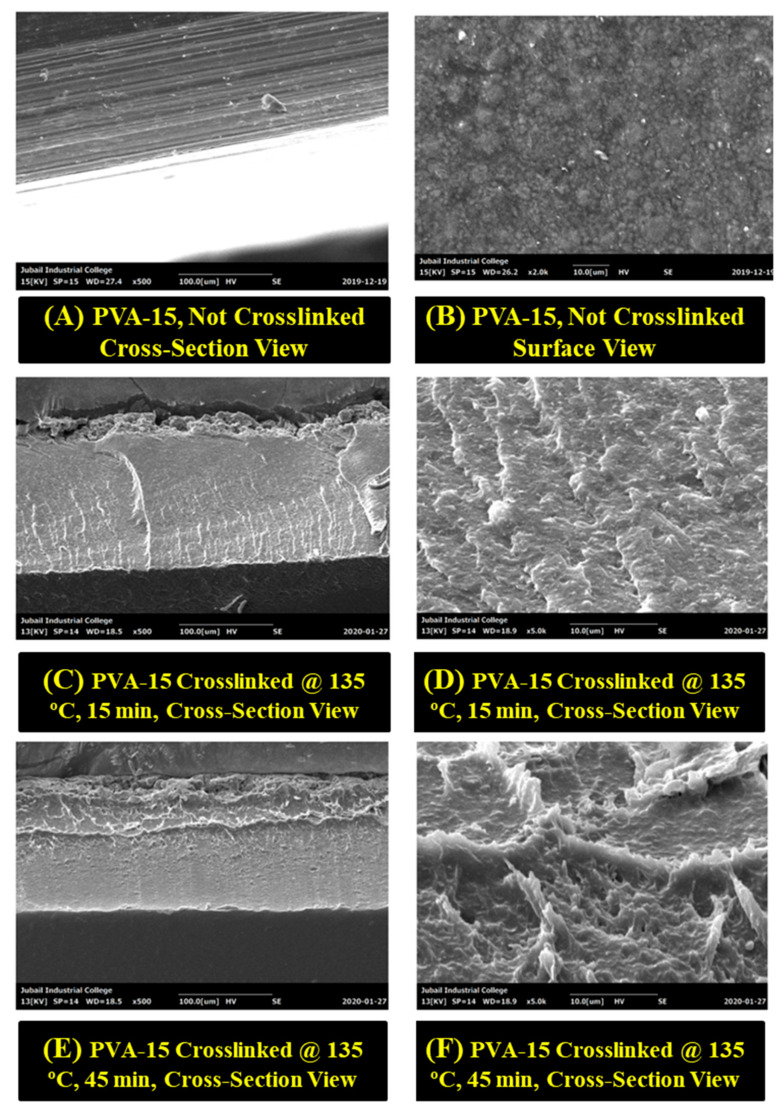
SEM analysis of the prepared PVA-15 NCFs with different magnifications.

The morphologies of the prepared PVA-15 NCFs are displayed in Figure 8. The non-crosslinked PVA-15 NCF revealed well-dispersed nanoclays inside the PVA matrix (Figure 8A) [55], and the film exhibits surface roughness due to the incorporation of the nanoclay microparticles (Figure 8B). The SEM images of the crosslinked PVA-15 NCFs provide further evidence of the well-dispersed nanoclays into PVA matrix as well as the absence of phase separation at the microscale level (Figure 8D,F). The morphology of the crosslinked NCFs exhibits higher dense structure when the crosslinking time was increased from 15 to 45 min, which suggests that nanoclays have participated in the crosslinking reaction, which agrees with the FTIR analysis results. It has been reported that the nanoclays embedded inside the PVA/MMT NCFs exhibit a randomly oriented structure when the content of the nanoclays is <30%, whereas layered structure is observed when the content of nanoclays suppresses 30% [91]. In the current study, the obtained morphologies of the nanoclays embedded inside the PVA matrix exhibit random oriented structure arrangement which agrees with the former literature report (Figure 8D,F).

### 3.5. Mechanical Properties of the NCFs

**Figure 9:**
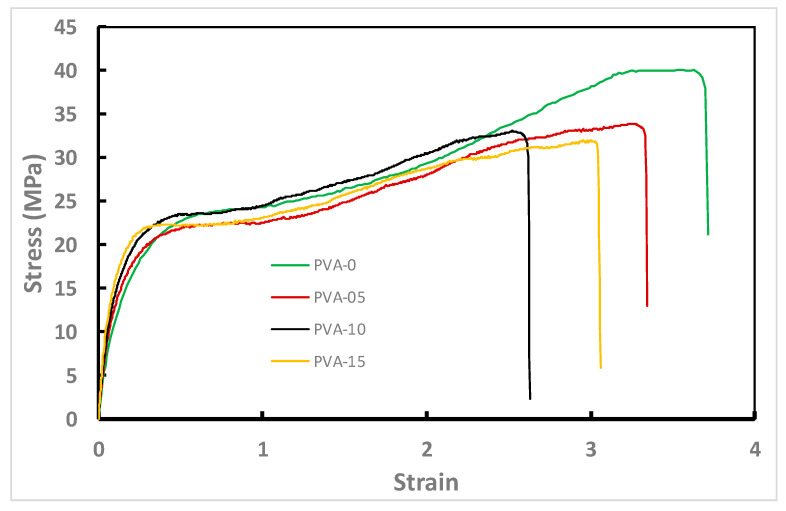
Typical stress–strain plots of PVA materials with different nano-clay content. All films were crosslinked at 135 °C for 45 min.

**Figure 10:**
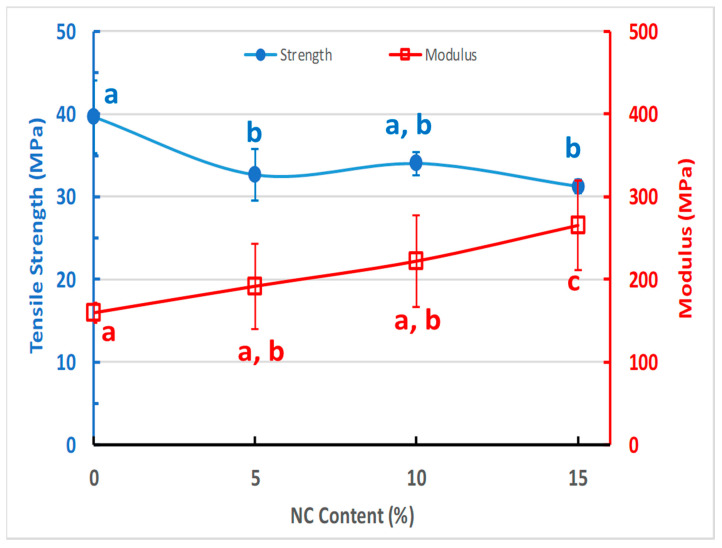
Tensile strength and modulus of elasticity (right axis) of PVA materials as a function of nano-clay content. Error bars indicate the standard deviation in the average test values of 5 samples. Values with the same letter in the same row do not differ statistically by Tukey’s test (*p* ≤ 0.05).

**Figure 11:**
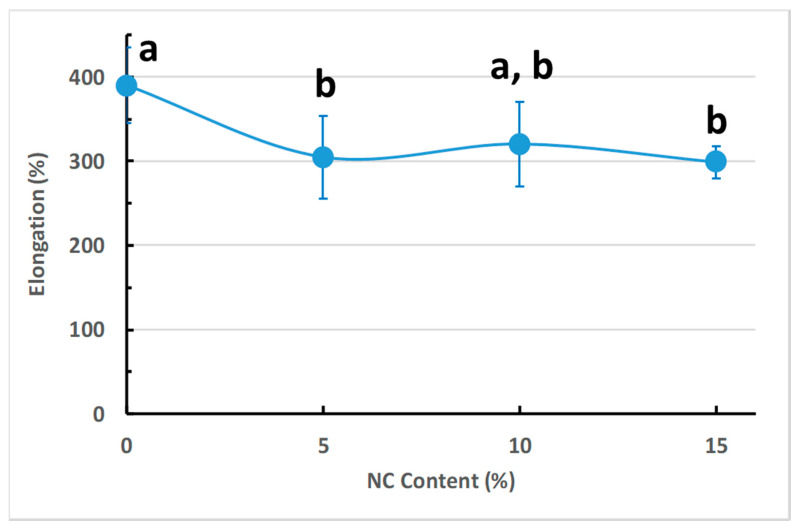
Percent elongation (ductility) of PVA materials as a function of nano-clay content. Error bars indicate the standard deviation in the average test values of 5 samples. Values with the same letter in the same row do not differ statistically by Tukey’s test (*p* ≤ 0.05).

Nowadays, the commercial production of packaging films demands superior mechanical performance due to the diversity of transportation and distribution methods [92,93]. Figure 9 represents typical tensile tests for the prepared NCFs with different nanoclay loadings. The addition of nanoclay to PVA material has resulted in a slight decrease of the tensile strength and ductility which could be attributed to the formation of a crosslinked network via covalent bonding between PVA matrix and the dispersed nanoclays [81] (Figure 10). Regardless, the reinforcing and stiffening effect of nanoclays can be inferred from the linear increase in the average modulus for the NCFs from 160 (PVA-0) to 265 MPa for the 15% nanoclay loading (PVA-15) (Figure 10). This increase is a good indication of the randomly distributed nanoparticles in the polymer matrix, as also shown in SEM micrographs (Figure 8D,F). The presence of plasticizer is known to have a negative effect on tensile strength and elastic modulus due to the decrease in the intermolecular van der Waals forces between polymeric chains and the consequent increase in their molecular mobility [93]. Regardless, the addition of glycerol as a plasticizer allowed for obtaining tough NCFs without scarifying their ductility at 15% nanoclay loading (Figure 11). The obtained NCFs exhibit suitable mechanical properties for food packaging applications as they possess average mechanical properties that are comparable to the commercial food packaging films made from low density polyethylene (LDPE), linear low density polyethylene (LLDPE), ethylene vinyl acetate copolymer (EVA) and ethylene vinyl alcohol copolymer (EVOH) (Table 2).

### 3.6. Optical Properties of the NCFs

**Figure 12:**
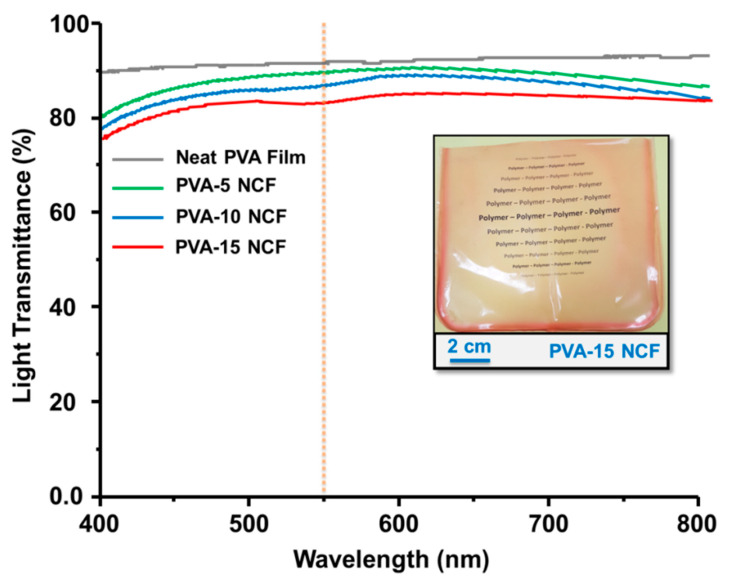
Transmittance for the prepared NCFs. The inset image shows the digital image for the PVA-15 NCFs placed on the top of yellow Xerox paper printed with “Polymer” words.

Figure 12 displays the transmittance properties for the prepared NCFs. Neat PVA film exhibits a transmittance of 85%. All NCFs exhibit high transparency with a light transmittance of ≈ 79–83%. The slight reduction in the optical transmittance is attributed to the light scattering by the embedded nanoclay platelets inside the polymer matrix.

**Figure 13:**
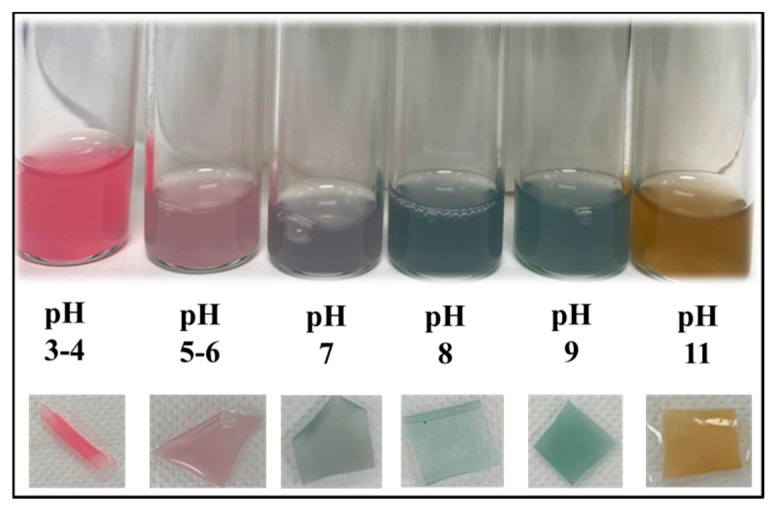
Digital images for the solutions (**top**) and NCFs (**bottom**) at different pH values.

Color parameters for the prepared NCFs at selected pH values are listed in Table 3. The lightness of the NCFs (L*) is almost constant and provides an indication about the absence of color degradation in both acidic and alkaline medium, which reflects the stability of the anthocyanin color at the entire pH range. The a* parameter has positive and high values in the acidic medium in the range of pH 3 to pH 6, where the NCF exhibits red color at pH 3–4 and pink color at pH 5–6 (Figure 13). When the medium became neutral at pH 7, the a* value decreased further but was still in the positive range, as the NCF exhibits a light purple color (Table 3). However, in alkaline medium from pH 8 to pH 9, the NCF exhibits a distinct green color, which is reflected from the recorded a* negative values (Table 3). The inflection points of the (a*) below and above pH 7 allows for using the NCFs as colorimetric pH-sensor and pH-indicative film for monitoring the food freshness/spoilage (Figure 13). This result is supported by the calculated values of ∆E in the range of ≈ 8–34, which indicates that the color change can be observed by the naked eye from neutral medium (pH 7) to acidic/alkaline pH ranges (Table 3). The clarity of the color change can be further enhanced by obtaining higher ∆E values as in the case when the transition occurs from pH 3 to pH 8–9. Hence, in this study, all the NCFs were cast at pH 3 with reddish color appearance, and used as pH-indicative films for providing fast response and distinctive change to bright green color at pH 8–9 (∆E ≈ 39–50) (Table 3). It is worth mentioning that both green and red colors are complementary colors and the transition from pink-reddish colors to greenish color derivatives was effective for observing and detecting different phenolic compounds with paper-based chemical sensor platform [96]. This can be seen from the highest color difference (∆E ≈ 50) for the transition from red at pH 3 to green at pH 9 as compared to the color difference (∆E ≈ 46) for the transition from red at pH 3 to yellow at pH 11 (Table 3).

### 3.7. Application of the NCFs as pH-Indicative Films for Food Packaging

**Figure 14:**
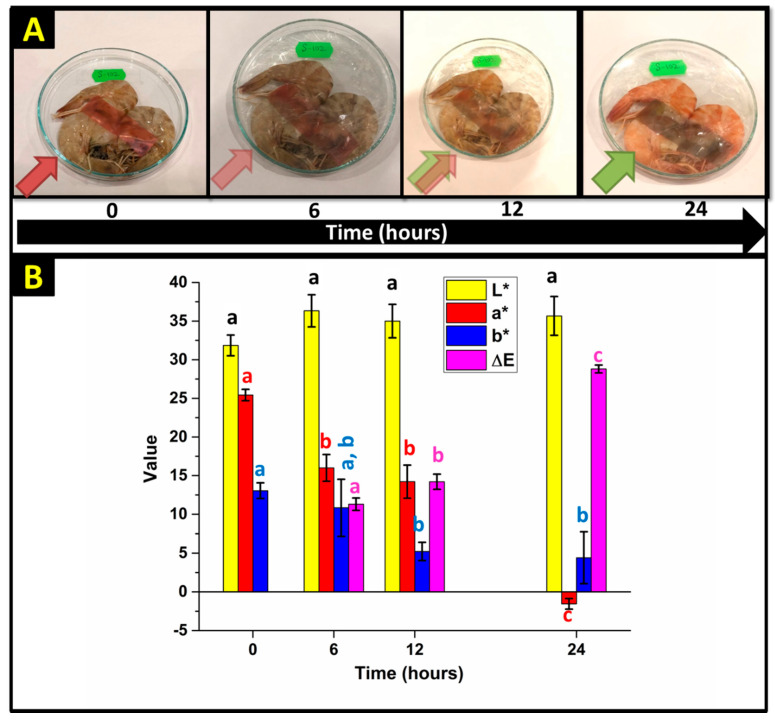
(**A**) Color change of PVA-15 NCFs in contact with shrimp samples exposed to air during the test period of 24 h. The added arrows indicate the color of the corresponding NCFs. (**B**) The corresponding values for the color parameters (L*, a* and b*) and color difference (∆E) for the NCF used in the shrimp spoilage test at different time intervals. The results are the means of three determinations ± standard deviation. Values with the same letter in the same row do not differ statistically by Tukey’s test (*p* ≤ 0.05).

Shrimp samples have been used as a model food for verifying the effectiveness of the pH-indicative sensor, as there is a strong correlation between the pH of the shrimp samples and their freshness [97]. The cast PVA-15 NCFs, crosslinked at 145 °C for 45 min, were applied as pH-indicative films for testing the freshness of shrimp samples (Figure 14A). Fresh shrimp samples were intentionally spoiled in ambient conditions to observe the successive color change of the pH indicative films during the testing period. Initially, the indicative films were red as they were prepared at pH 3. After 6 h, the films turned into light pink color, which indicates that the pH has increased to pH ≈ 5–6, with color difference (ΔE = 11.3) (Figure 14B). This is attributed to the release of volatile nitrogenous compounds (e.g., ammonia and triethylamine) from shrimp’s proteins when they start to get spoiled due to bacterial growth and microbial degradation [98]. The color change increased slightly after twelve hours due to the presence of mixed areas with both light pink and light green colors, as reflected from the calculated color difference (ΔE ≈ 14.2). The total spoilage of the shrimp samples was confirmed after 24 h, which can be inferred from the appeared bright and distinctive olive green color (pH 8–9) of the pH-indicative film, with high difference in color change (ΔE ≈ 29).

## 4. Conclusions

The prepared NCFs with 15% nanoclay have shown very good film-forming properties, as revealed from SEM analysis, high transparency, very low solubility, and low swelling properties compared to many precedent literature reports. The elastic modulus was improved for the NCFs, which showed comparable mechanical properties to other commercially available packaging films. The stability of the indicator dye inside the films was improved during the crosslinking treatment.

The NCFs allowed visual detection for the gradual transition from acidic to alkaline medium. The obtained NCFs showed flame-retardant behavior, especially after sufficient crosslinking treatment, which resulted in lowering both the enthalpy of melting (∆H_m_) and the enthalpy of degradation (∆H_d_). Thus, the fabricated NCFs are envisaged as multifunctional, green, and sustainable alternatives to those commercially available petroleum-based thermoplastics for smart and safe packaging applications.

## Figures and Tables

**Table 1:** Analysis results from DSC measurements *.

	T_g_ (°C)	T_m1_ (°C)	ΔH_m1_ (J/g)	T_m2_ (°C)	ΔH_m2_ (J/g)	T_d_ (°C)	ΔH_d_ (J/g)	*χ*_c_ (%)
Neat PVA	76.2 ± 1.1 ^b^	194.0 ± 0.1 ^b^	34.4 ± 0.8 ^a^	-	-	310.0 ± 1.5 ^b^	165.0 ± 2.9 ^a^	25 ± 0.8 ^a^
PVA-15, Not crosslinked	81.1 ± 0.7 ^a^	199.0 ± 0.08 ^a^	25.3 ± 0.6 ^b^	232.6 ± 1.5 ^a^	15.6 ± 1.2 ^a^	326.0 ± 0.6 ^a^	55.0 ± 1.4 ^a^	18 ± 0.6 ^b^
PVA-15, Crosslinked at 135 °C, 45 min	Not detected	199.2 ± 0.04 ^a^	14.2 ± 0.7 ^c^	232.8 ± 1.3 ^a^	7.6 ± 0.7 ^b^	326 ± 0.4 ^a^	26.2 ± 0.8 ^c^	10 ± 1.2 ^c^

* Results are the means of three determinations ± standard deviation. Values with the same letter in the same row do not differ statistically by Tukey’s test (*p* ≤ 0.05).

**Table 2:** Mechanical properties for selected commercial polymer films.

	Elongation at Break (%)	Tensile Strength at Break (MPa)	Modulus (MPa)	Reference
LDPE	130–540	22–26	240–290	[94]
LLDPE	570–850	35–50	190–220	[94]
EVA	530	31	48	[94]
EVOH	13–16	24–42	3550–5200	[95]
PVA-15 NCFs	310 ± 20	31 ± 1	265 ± 45	This study

**Table 3:** Color parameters at different pH values for PVA-15 NCF crosslinked at 135 °C for 15 min *.

pH	L *	a *	b *	∆E
∆E is calculated with reference to pH = 7				
pH 3	59.65 ± 0.41 ^c^	37.50 ± 0.46 ^a^	2.53 ± 0.05 ^e^	32.73 ± 0.18 ^e^
pH 6	62.81 ± 0.30 ^a^	18.60 ± 0.30 ^b^	6.42 ± 0.20 ^d^	14.60 ± 0.22 ^g^
pH 7	57.69 ± 0.10 ^d^	5.25 ± 0.05 ^d^	8.03 ± 0.11 ^c^	0 ^i^
pH 8	62.00 ± 0.11 ^b^	−1.40 ± 0.12 ^e^	8.11 ± 0.09 ^c^	7.94 ± 0.10 ^h^
pH 9	54.27 ± 0.21 ^e^	−11.27 ± 0.07 ^f^	9.28 ± 0.02 ^b^	16.94 ± 0.16 ^f^
pH 11	57.83 ± 0.07 ^d^	12.92 ± 0.02 ^c^	41.11 ± 0.10 ^a^	33.88 ± 0.06 ^d^
∆E is calculated with reference to pH = 3				
pH 8	61.80 ± 0.13 ^b^	−1.37 ± 0.03 ^e^	8.03 ± 0.05 ^c^	39.31 ± 0.05 ^c^
pH 9	54.23 ± 0.06 ^e^	−11.28 ± 0.07 ^f^	9.24 ± 0.06 ^b^	49.59 ± 0.01 ^a^
pH 11	57.84 ± 0.15 ^d^	13.00 ± 0.10 ^c^	41.3 ± 0.03 ^a^	45.85 ± 0.05 ^b^

* Results are the means of three determinations ± standard deviation. Values with the same letter in the same row do not differ statistically differ by Tukey’s test (*p* ≤ 0.05).
